# Non-volatile reconfigurable spin logic functions in a two-channel Hall bar by spin–orbit torque-based magnetic domains and directional read current

**DOI:** 10.1038/s41598-023-38580-1

**Published:** 2023-07-18

**Authors:** JeongHun Shin, Jeongwoo Seo, Saegyoung Song, WooJong Kim, Da Seul Hyeon, JinPyo Hong

**Affiliations:** 1 Division of Nanoscale Semiconductor Engineering, Hanyang University, Seoul, 133-791 South Korea; 2 Novel Functional Materials and Device Laboratory, Department of Physics, Research Institute of Natural Science, Hanyang University, Seoul, 133-791 Korea

**Keywords:** Magnetic properties and materials, Spintronics, Electronic and spintronic devices

## Abstract

A long-standing goal of CMOS-based logic devices is to meet the needs of key markets, including ultralow-power operation and high operation speed, along with the continuing miniaturization of the architecture. However, despite significant progress in their development, conventional CMOS-based devices still suffer from drawbacks such as introducing large unintended leakage currents and volatile behavior. Thus, reconfigurable logic gates based on magnetic domain (MD) have emerged as a highly promising option because they offer fast operation speeds, nonvolatility, and diverse logic functions in a single-device configuration. Here, we address multiple reconfigurable MD logic gates in a single two-channel Hall bar device by varying the voltage-driven read-current directions and selecting a non-inverting or inverting comparator in W/CoFeB/MgO/Ta stacks. The non-volatile MD switching behavior induced by spin–orbit torque significantly affects our logic gate functions, which are not necessarily synchronized to a single clock. By adapting MD switching by spin-orbit torque and anomalous Hall effect voltage outputs, we identified eight reconfigurable logic gates, including AND, NAND, NOR, OR, INH, Converse INH, Converse IMP, and IMP, in a single device. These experimental findings represent a significant step forward in a wide range of MD-based logic applications in the near future.

## Introduction

Spin-based devices based on the manipulation of the spin degree of freedom in magnetic systems are of considerable interest as one of the most reliable options for providing numerous advantages, such as high dynamic speed, low leakage current, thermal stability and non-volatile memory, compared to conventional silicon-based complementary metal oxide semiconductors (CMOS)^1^. Among the most prominent spin devices is the spin–orbit torque magnetic random-access memory (SOT-MRAM), which is beneficial for a relatively fast driving speed, low power consumption, and durable performance^2^. Therefore, in recent years, the spin–orbit torque (SOT) induced by diverse heavy metals (HM) such as Ta and W under bias has gained importance as a promising alternative for next-generation spin devices^3–7^.

To ensure such promises, a few spintronic devices based on the SOT effect are spin-based adder subtractors, neuromorphic devices including half skyrmions, and logic devices^8–12^. The Dzyaloshinskii-Moriya interaction (DMI), an important magnetic surface coupling effect, is crucial in spin-based logic devices utilizing the SOT effect and domain wall motion^13–18^. The DMI arises from spin–orbit coupling at the interface between a magnetic layer and a non-magnetic heavy metal layer, leading to chiral magnetism and the formation of unique spin textures such as skyrmions^19^. The chiral spin structure has been utilized for logic operations based on chirally coupled nanomagnets or domain wall motion through chirality switching^20^. These findings emphasize the significance of considering the DMI when designing and implementing spin-based logic devices^20^.

In particular, SOT-based reconfigurable logic devices are expected to provide solutions for ultralow-power, high-speed, high-density, and non-volatile systems. These devices can also perform multiple logic operations in a single device frame, enhancing their efficiency compared to conventional logic devices^21–29^. For example, numerous studies of spin-based reconfigurable logic devices have also reported successful logic operations using skyrmion dynamics, magnetic tunnel junctions, and chirality-based vortex domain walls^30–34^.

Among the various approaches for spin reconfigurable logic devices, those employing current-induced magnetic domain (MD) switching have also attracted considerable interest as basic building blocks for advanced logic component deployments^21, 24^. Experimental demonstrations of MD wall-based logic components using magnetic tunnel junctions have been reported^35, 36^. Recently, researchers have investigated the performance of a reconfigurable MD logic gate by manipulating the anomalous Hall effect (AHE) voltage output signals conjugated by the SOT effect. Although MD logic gates by AHE voltage have been reported previously, they still seek to exploit the practical implementation of multiple reconfigurable logic gate in a single device configuration and use the advantage of non-volatile behavior^22, 24, 25^.

In this letter, we introduce the implementation of eight reconfigurable spin-logic gates using SOT-driven MD switching in a single two-channel Hall bar frame. The AHE voltage was systematically monitored by employing various circuit connections for the logic gate. One concept of MD logic gates involves manipulating SOT current directions in a two-channel Hall bar configuration under bias, eliminating the need for time synchronization between inputs owing to stability of the MD. The second concept involves the manipulation of the output voltage by varying the read current directions in a two-channel Hall bar configuration under the same bias. We describe reconfigurable logic gates such as AND, NAND, NOR, OR, Converse INH, Converse IMP, INH and IMP in a single MD logic gate by selecting a non-invert and invert comparator under a fixed external magnetic field.

## Results and discussion

**Figure 1:**
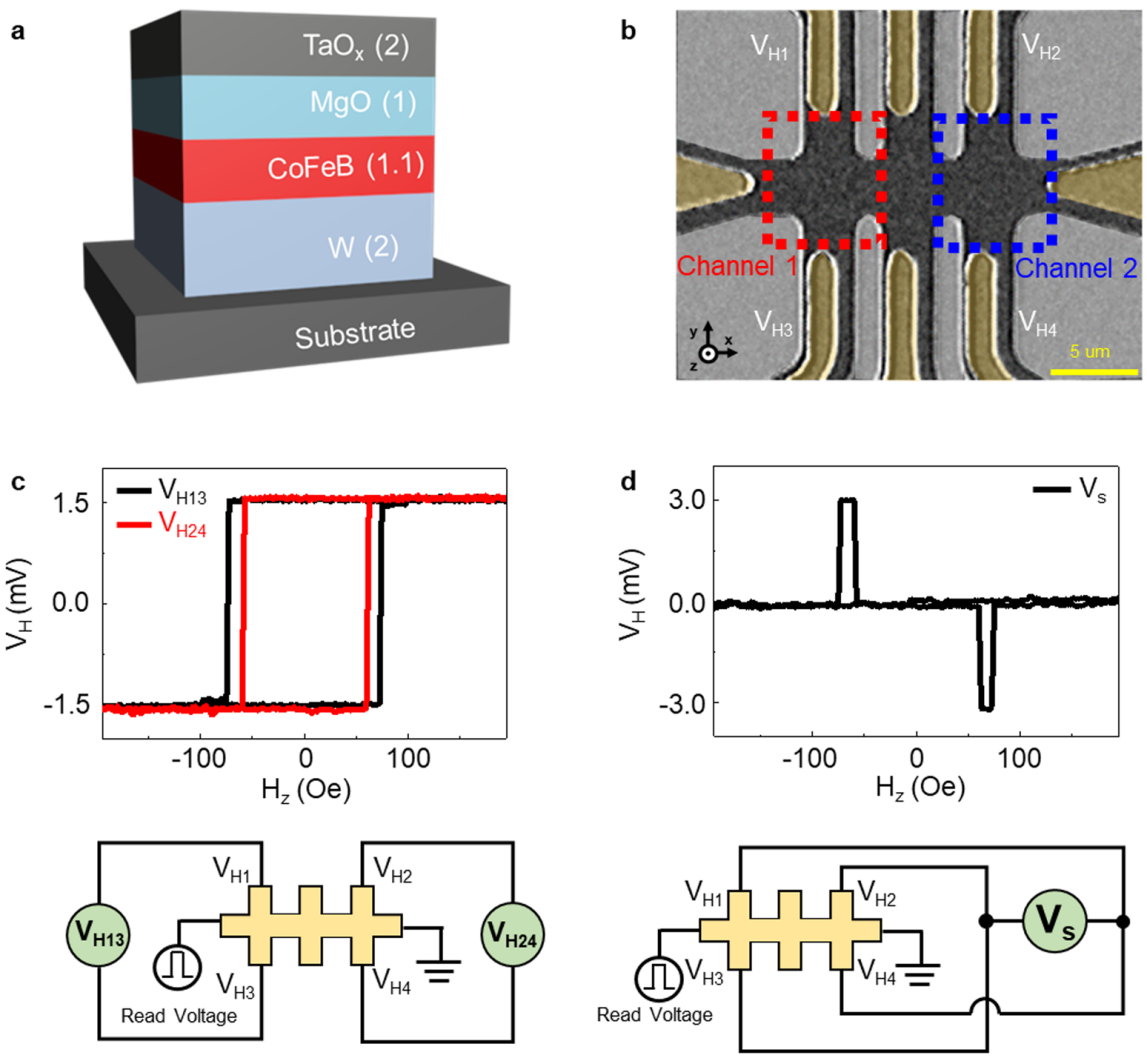
Sample schematic and magnetic characteristics of two-channel Hall bar. (**a**) Device stacks of substrate/W (2 nm)/CoFeB (1.1 nm)/MgO (1 nm)/TaO_x_ (2 nm) layers with perpendicular magnetic anisotropy. (**b**) Optical images of the two-channel Hall bar device, highlighting the AHE voltage electrodes leveled by V_H1_ ~ V_H4_. (**c**) Plot of Hall voltage V_H_ versus out-of-plane magnetic field (H_z_). The black line represents the AHE voltage of Channel 1 (V_H13_), which was monitored by connecting V_H1_ and V_H3_ to a voltage meter, whereas the red line corresponds to the AHE voltage of Channel 2 (V_H24_). (**d**) Subtracted Hall voltage (V_s_) versus out-of-plane magnetic field (H_z_), defined as the difference between the AHE voltages in channels 1 and 2. The electrical circuit schematic for the measurement is shown in the lower part of Figures (**c**) and (**d**).

We fabricated sample stacks of substrate/ W/2 nm Co_20_Fe_60_B_20_/1.1 nm MgO/1 nm TaO_x_/2 nm with perpendicular magnetic anisotropy, as shown in Fig. 1a. The films were deposited on 200-nm-thick thermally oxidized Si substrates by magnetron sputtering at room temperature under a base pressure of < 7 × 10^–9^ Torr and an Ar pressure of 3 mTorr. Post-annealing was conducted at 350 °C for 30 min under vacuum conditions of < 1 × 10^–6^ Torr with a 3 T perpendicular magnetic field. The two-channel Hall bar was patterned into 5 μm width using photolithography and Ar ion milling, followed by an oxygen plasma ashing process for 2 min with 80 W radiofrequency power to remove residual photoresist during ion-milling procedures.

Figure 1b depicts a representative Hall bar configuration consisting of channels 1 and 2, highlighted in red and blue dashed boxes, respectively. The AHE voltage (V_H_) is mainly determined by the z-component of net magnetization. Four AHE voltage electrodes (V_H1_, V_H2_, V_H3_, and V_H4_) are used to monitor V_H_. The following equation describes the relationship between V_H_ and read current (J_read_):

1 $${V}_{H}= \frac{{R}_{s}{M}_{s}{J}_{read}}{{t}_{FM}}{m}_{z}$$

where R_s_, M_s_, and t_FM_ represent the anomalous Hall coefficient^37^, saturation magnetization of the ferromagnetic metal (FM) layer, and thickness of the FM layer, respectively. V_H_ is proportional to MD (m_z_) and J_read_ directions. The value of the AHE voltage is determined by the sign of m_z_ for a given J_read_. The AHE voltage of each channel is probed using V_H1_, V_H2_, V_H3_, and V_H4_ in the Hall bar structure. Figure 1c shows the AHE voltages (V_H13_, V_H24_) of channels 1 (black line) and 2 (red line) as a function of the applied out-of-plane magnetic field. The presence of hysteresis loops ($\left|{H}_{c}\right|$ = 60 and 75 Oe) indicated the preserved PMA characteristics in the patterned channels. The slight difference in H_c_ between both channels seems to be due to the presence of unintended defect sites in the channel 2 allowing for the rapid MD generation, compared to that of channel 1. Figure 1d shows the circuit schematic of the Hall bar structure and the voltmeter (green) used to determine a subtracted AHE voltage (V_s_), where the V_s_ is defined as the difference in the AHE voltages between channels 1 and 2 in one measurement step and can be calculated as follows:

2 $${V}_{s}= {V}_{H13}-{V}_{H24}=\left({V}_{H1}-{V}_{H3}\right)-\left({V}_{H2}-{V}_{H4}\right)=\left({V}_{H1}+{V}_{H4}\right)-({V}_{H2}+{V}_{H3})$$

The MDs of the two-channel are aligned in opposite directions between ± H_c_ = 60 and 75 Oe, as seen in Fig. 1c. The opposite direction of MD produces a voltage difference of V_s_ =  ±3 mV. The V_s_ is zero (V_s_ = 0) when the MD is aligned in the same direction. It is well-known that spin accumulation occurs at the interfaces when an unpolarized charge current flows through a HM layer with a large spin–orbit coupling. The accumulated spins diffuse into the adjacent FM, leading to MD switching, which is called SOT switching. The x-axis current induces spin polarization along the y-axis. The external magnetic field (H_x_) and SOT current (J_n_) induce MD state (m_z,n_) alignment, as is evident in previous results^38^. The positive spin Hall angle (θ_sh_) of W ^39, 40^ determines the MD switching, as indicated by the following equation:

3 $${J}_{n} \cdot {H}_{x}>0\Rightarrow +{m}_{z,n}, {J}_{n} \cdot {H}_{x}<0\Rightarrow -{m}_{z,n}$$

As discussed above, a sufficient SOT current in the W layer of the PMA sample allows for up- or down-MD switching under magnetic field H_x_. The DMI effect of the logic gate has an impact on SOT and MD switching. Although the measurement of DMI value has not been conducted in our work, it is expected that the DMI value on our logic gate would be similar to the result described by other previous work^18^ since the device structure of other work is similar to the our device. Basically, our logic gate focuses on the reconfigurable logic operation based on the SOT and AHE voltage.

**Figure 2:**
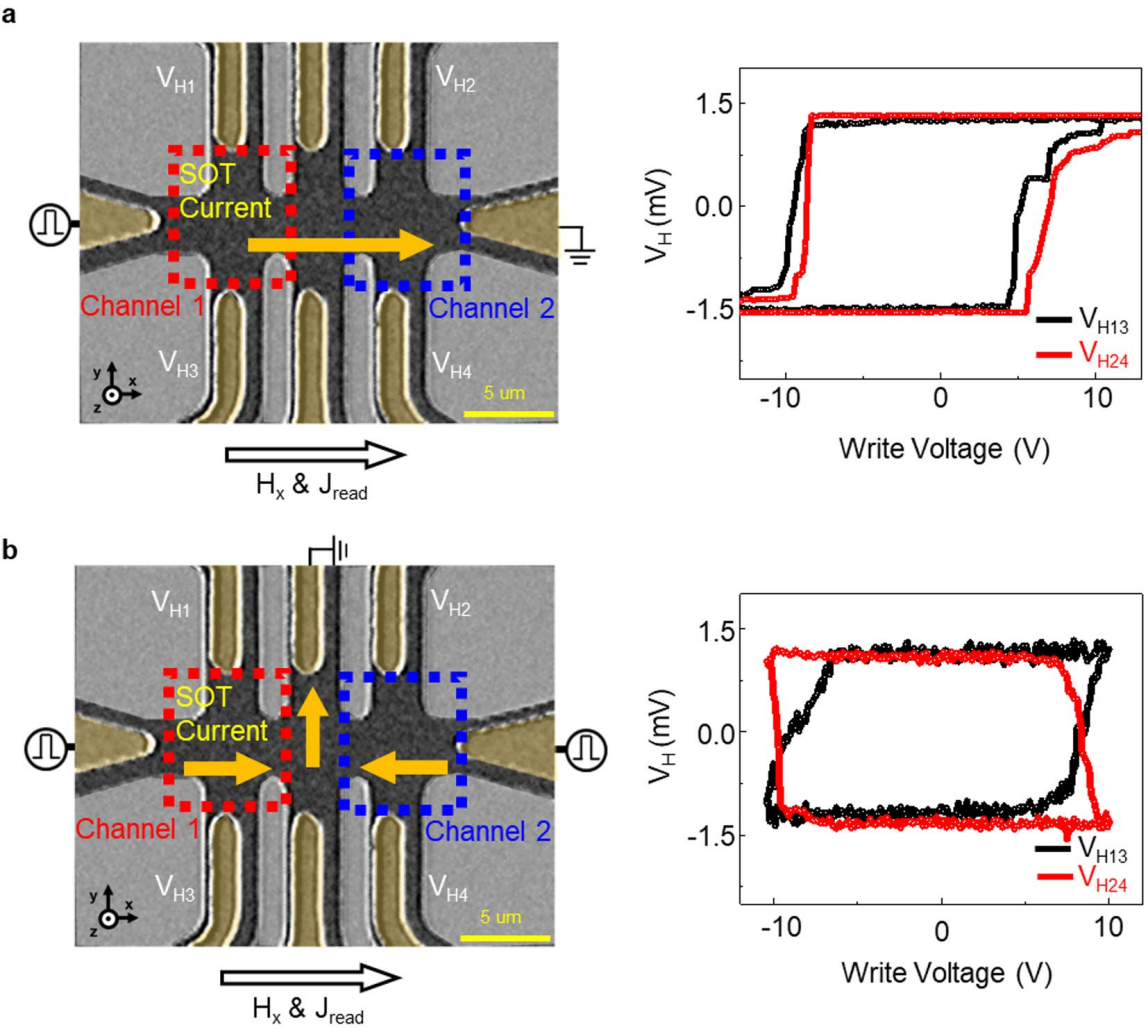
Microscope images and magnetic domain switching characteristics of a two-channel Hall bar via SOT current. (**a**) Optical image of the SOT current directions (yellow arrows) and AHE voltages versus write voltage in the same direction in a two-channel Hall bar device. (**b**) Optical image of two opposite SOT current directions (yellow arrows) and AHE voltages versus write voltage. The V_H13_ and V_H24_ curves in (**a**) and (**b**) represent the independently switched AHE voltages of channels 1 and 2, respectively, as monitored by the read current flowing along the x-axis with a read voltage of 1 V.

To demonstrate the SOT switching in a two-channel Hall bar, a write voltage pulse is applied from the left voltage source connected to channel 1 and the SOT current flows through channels 1 and 2, as shown in Fig. 2a. The write voltage pulse duration and H_x_ are 100 ms and 100 Oe, respectively. To ensure individual MD switching in the two-channel Hall bar by SOT current, MD switching is tested based on the voltage source position (Fig. 2b). The results demonstrate that when a write voltage pulse is applied from the left or right voltage source, the MD switching of channels 1 and 2 occurs in the opposite direction. The AHE voltage is attained at 1 V of read voltage after the application of the write voltage pulse. In this measurement, the write and read voltage pulses of 10 and 1 V correspond to the switching current densities of 1.0 × 10^7^ A/cm^2^ at H_x_ = 100 Oe and 1.0 × 10^6^ A/cm^2^, respectively. In our work, the logic gate operates with a write voltage of 10 V and a current of 3 mA, with a pulse width of 100 ms. This results in an energy consumption of 3 mJ and an operation speed of 100 ms. However, when we estimate energy consumption and operation speed for our downscaled devices based on the parameters mentioned in the previous study^41^. By using a current density of 2.01 × 10^12^ A/m^2^ and a pulse width of 5 ns under a magnetic field of 200 Oe, the energy consumption is estimated to be 16 nJ, which is significantly reduced compared to the previous value of 3 mJ. The operation speed is estimated to be 5 ns. The estimations in downscaled device suggest a possible potential for the achievement of lower energy consumption and faster operation in our logic gate.

**Figure 3:**
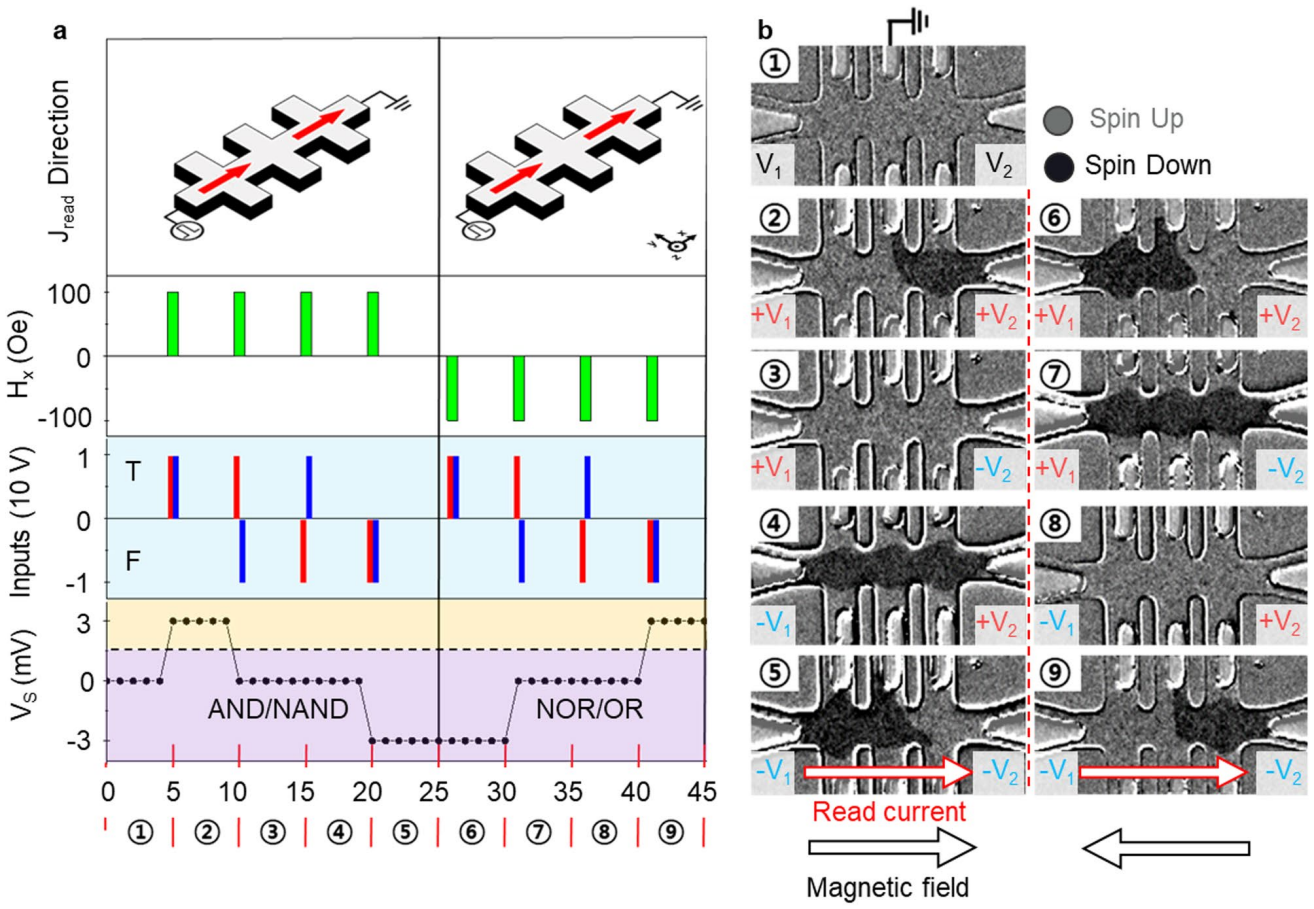
Reconfigurable logic gate by inputs and their corresponding MOKE image sequence. (**a**) Logic gate output for the write input parameters at ±V_1_ and ±V_2_ (±10 V), indicated by red and blue bars. Sections ① ~ ⑤ displays V_s_ values of +3 mV (high state), −3 mV (low state), and 0 mV (middle state) with an external magnetic field H_x_ and read currents oriented rightward along the x-axis. Sections of ⑥ ~ ⑨ show states with symmetrically varying H_x_ b) Sequential MOKE images of MDs in each channel for inputs (+V_1_, +V_2_), (−V_1_, +V_2_), (+V_1_, −V_2_), and (−V_1_, −V_2_). The bright and dark areas represent the upward- and downward-pointing spins in the MDs, respectively.

To provide more details on the reconfigurable logic gates, the basic structure of the gate consists of a two-channel Hall bar composed of one logic output via four V_Hn_, two logic inputs, and one read bias. The logic gate functions illustrate that the three parameters are conducted with different logic-input configurations (V_1_, V_2_), H_x_, and read voltage (V_read_ = 1 V, J_read_ = 1.0 × 10^6^ A/cm^2^) in the same structure, as shown in Fig. 3a. Four logic-input configurations of ‘TT,’ ‘TF,’ ‘FT,’ and ‘FF’ are identified by applying ± 10 V amplitude and 100 ms pulse width. The red (V_1_) and blue (V_2_) bars mark the logic-input configurations (Fig. 3a). One significant advantage of this work is that time synchronization for MDs is not required owing to their non-volatile behaviors. V_1_ is first applied, followed by V_2_ after an interval of 10 ms, and a small read voltage pulse (1 V, 100 ms) is applied, at the end of which the AHE voltage is observed. In Fig. 3a, each interval where logic inputs are applied is represented by the displayed ⓝ, and the corresponding MD in each subtracted Hall voltage state is illustrated by the MOKE images (Fig. 3b). Sections ① ~ ⑤ illustrate the logic gate behavior under an external magnetic field H_x_, where a read current J_read_ is applied rightward along the x-axis. In Fig. 3a, the dashed lines in the V_s_ output plot are divided into orange and purple regions based on V_s_ = 2 mV. The logic gate between AND and NAND can be reconfigured by selecting a comparator reference at 2 mV. For example, when V_s_ > 2 mV (orange color), the logic output is ‘T,’ allowing for the AND gate operation, defined by a non-inverting comparator. Conversely, when V_s_ < 2 mV (purple color), the logic output is ‘T,’ defined by an inverting comparator, permitting the NAND logic gate to be achieved. Sections of ⑥ ~ ⑨ represent the logic gate behaviors under a H_x_ leftward along the x-axis and J_read_ rightward along the x-axis. Based on Eqs. (1) and (3), the switching of the MD is reversed by an external magnetic field, resulting in an inversion of the AHE voltage. The corresponding results are implemented in the reconfigurable logic gates of the NOR or OR (Fig. 3b). Additionally, based on Eq. (1), the reconfigurable logic gates for NOR or OR are implemented by applying J_read_ leftward along the x-axis and H_x_ rightward along the x-axis. (Various MOKE images of MD switched by inputs are provided in supplementary Fig. S1).

**Figure 4:**
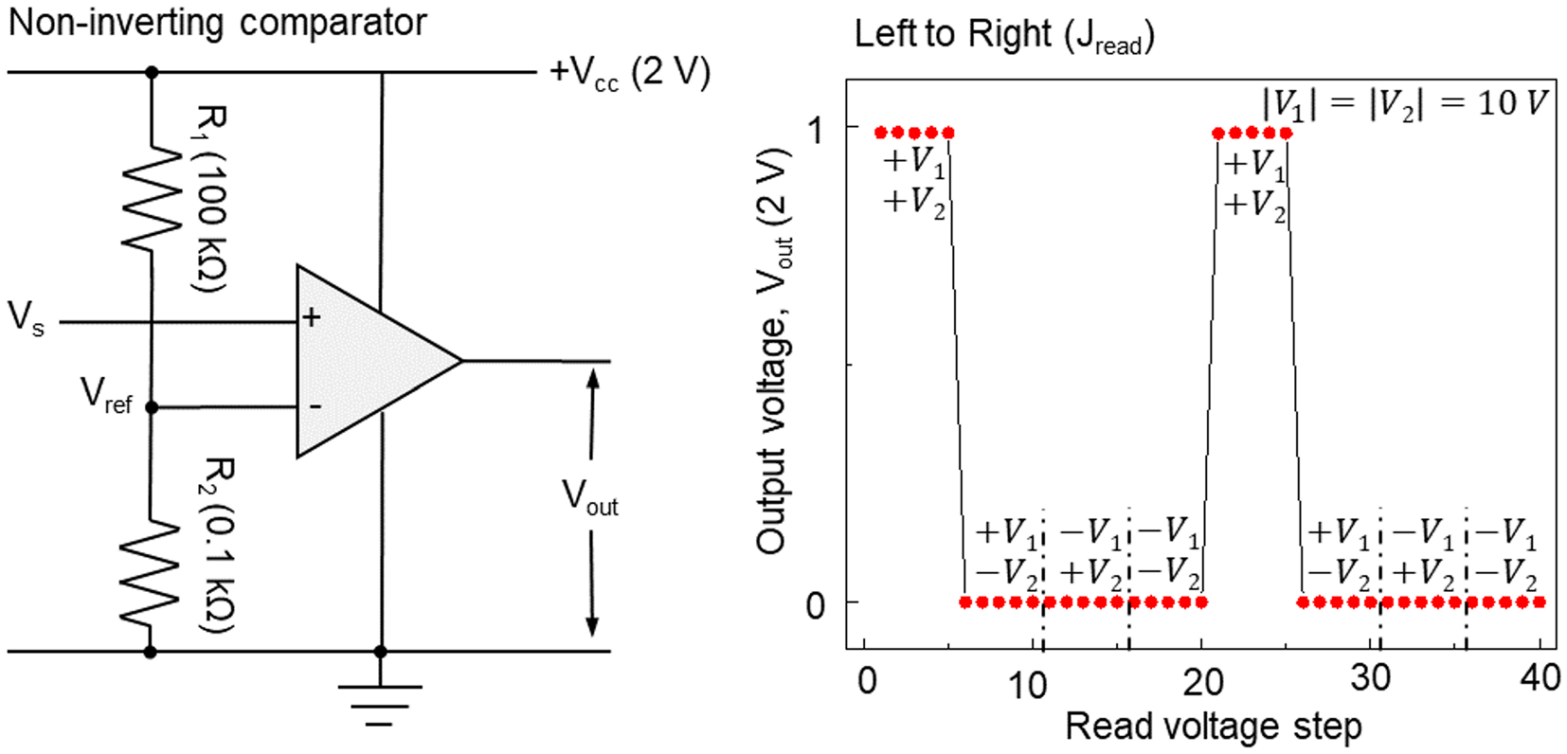
Comparator circuit schematic and V_out_ curves by non-inverting comparator. Op-amp comparator circuits with non-inverting configurations, where the circuit uses a comparison voltage V_ref_ of 2 mV to produce logic outputs (V_out_) in a non-inverting comparator circuit. The input voltages of both |*V*_1_| and |*V*_2_| have the same amplitude of 10 V and are used to switch the MD states. The positive and negative amplitudes of *V*_1_ and *V*_2_ correspond to the True and False, respectively. The read current, measured from left to right, is proportional to the voltage difference between the two inputs.

Figure 4 illustrates the implementation of logic gates using a non-inverting operational amplifier comparator circuit. The following equation determines the reference voltage (V_ref_) based on the voltage drop.

4 $${V}_{ref}=\frac{{R}_{2}}{{R}_{1}+{R}_{2}}*{V}_{cc}=\frac{0.1k}{100k+0.1k}*2\cong 2 mV$$

The function of a comparator is that if the positive terminal of the op-amp is larger than the negative terminal, the output signal is amplified, and if it is smaller, the output signal is reduced. The non-inverting comparator compares the values between V_s_ and V_ref_ to convert V_s_ into an output (V_out_). Figure 4 shows a non-inverting comparator circuit and corresponding V_out_. V_s_ is connected to the positive terminal, while V_ref_ is connected to the negative terminal of the op-amp. The operation of a non-inverting comparator is as follows; when V_s_ is larger than V_ref_, V_out_ is amplified to 2 V. Conversely, when V_s_ is smaller than V_ref_, V_out_ is attenuated to 0 V. By utilizing the resulting V_out_ from the non-inverting comparator, the two-channel Hall bar can effectively implement an AND gate.

The voltages V_1_ and V_2_ are applied at every five read-voltage steps. The absolute amplitudes of V_1_ and V_2_ are 10 V, corresponding to a switching current density of 1.0 × 10^7^ A/cm^2^ at H_x_ = 100 Oe. The positive and negative amplitudes correspond to ‘True’ and ‘False’ values, respectively. V_s_ is measured by applying J_read_ (1.0 × 10^6^ A/cm^2^) flowing left to the right along the channel. (The circuit and experimental result related to the inverting comparator are provided in Supplementary Fig. S2.)

**Figure 5:**
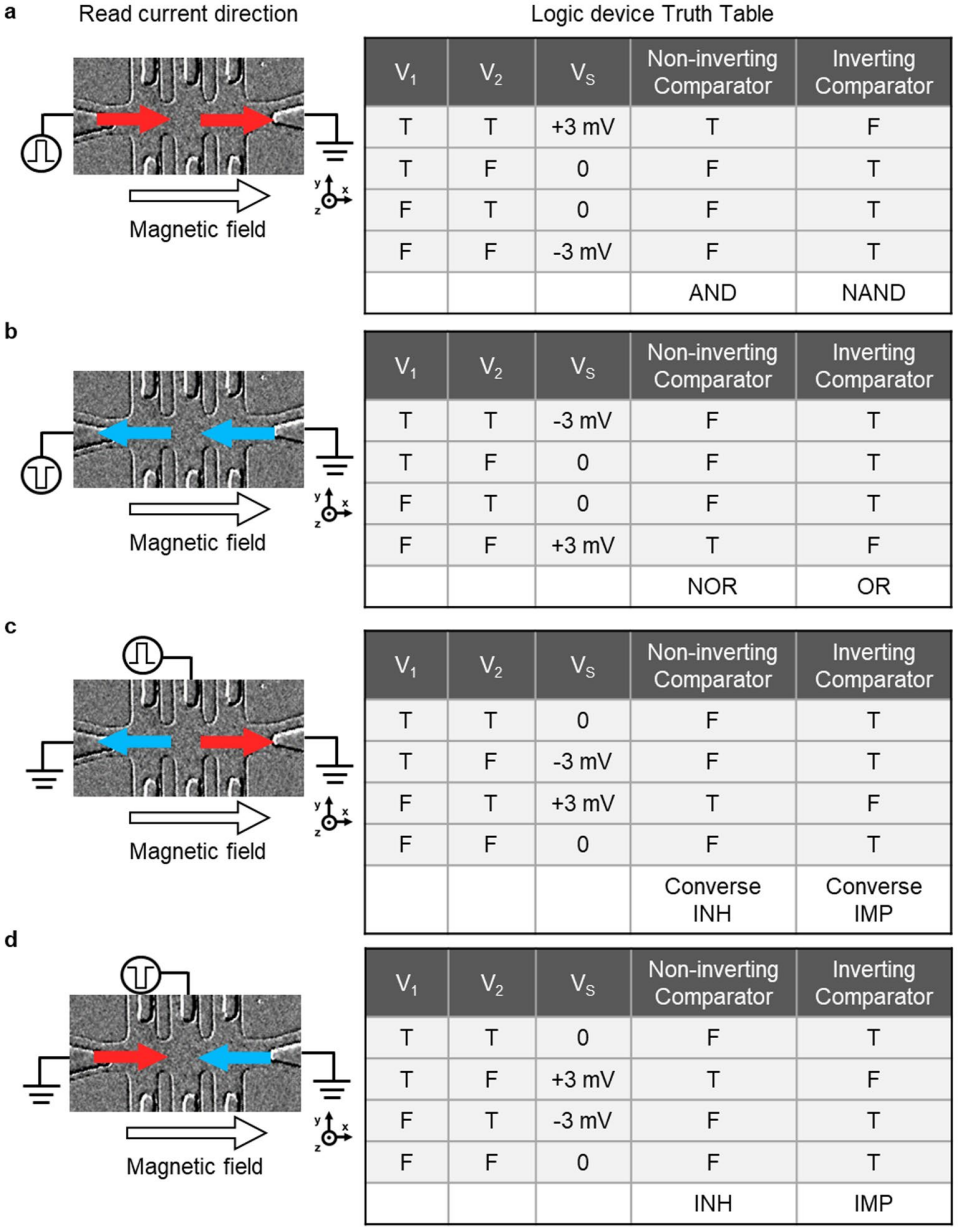
Schematic of various read current directions and their corresponding truth table. (**a**) Optical image of the read current direction (+x, +x) and its corresponding truth table. The logic gate is AND or NAND. (**b**) Optical image of the read current direction (−x, −x), identifying logic gate NOR or OR. (**c**) Optical image of the read current direction (−x, +x), reflecting logic gate Converse INH or Converse IMP. (**d**) Optical image of the read current direction (+x, −x), representing logic gate the INH or IMP. The red and blue arrows indicate that the read currents flowed toward the positive and negative x-axis at the same time, respectively. The all-logic gates are determined by selecting a non-inverting or inverting comparator.

Figure 5 exhibits the analysis of the eight logic gates (AND, NAND, NOR, OR, Converse INH, Converse IMP, INH, and IMP) from the two-channel Hall bar by separately adopting different J_read_ directions in a fixed H_x_. To determine the logic gate, the voltage source is connected to the left of channel 1 for the AND, NAND, NOR, and OR logic gates and to the center of channels 1 and 2 for Converse INH, Converse IMP, INH, and IMP, respectively. Before determining the logic gate using the read current, the MD is switched by applying inputs to the logic gate. Equation (3) is used to determine the direction of the MD based on H_x_ and J_n_. If H_x_ and J_n_ flow in the same direction, the MD is directed upward. If H_x_ and J_n_ flow in different directions, the MD is directed downward. H_x_ is applied rightward along the x-axis for all logic gates. When J_n_ flows rightward along the x-axis, the MD points up; when J_n_ flows leftward along the x-axis, the MD points down. Therefore, the MD of channel 1 is switched up when injected with J_1_ flowing rightward along the x-axis, induced by a positive V_1_, whereas the MD switching of channel 2 is obtained in the opposite direction because of the position of the write voltage source on the opposite side of the Hall bar.

Specifically, the MD of channel 2 is switched up when injected with J_2_ flowing rightward along the x-axis, as induced by a negative V_2_. Notably, the MDs of channels 1 and 2 can be switched differently even with the same voltage amplitude. The same MD switching results are obtained for all logic gates under the same input and external magnetic fields. Each of V_1_ and V_2_ inputs is denoted with ‘T’ and ‘F’ in case of positive and negative voltages. After applying the inputs mentioned above, reconfigurable logic gates are operated using various read current directions, as shown in the truth table in Fig. 5. Based on Eqs. (1), the direction of J_read_ can manipulate the AHE voltage of the channels. Figure 5a illustrates the implementation of AND/NAND logic gates using the read current flowing rightward along the x-axis in both channels. Figure 5b shows the NOR/OR logic gate caused by read currents flowing leftward along the x-axis in both channels. Figure 5c shows the Converse INH/ Converse IMP logic gate, which is observed by the read currents leftward and rightward along the x-axis for channels 1 and 2, respectively. When the MDs of both channels point in the same ± z directions, the AHE voltage of channel 1 is inverted, resulting in an output (V_s_) of ∓ 3 mV. (For more details on the correlation between V_s_ according to the read current polarity and amplitude, see Supplementary Fig. S3) Fig. 5d shows the INH/IMP logic gates under the read currents flowing in the right and left channels along the x-axis for channels 1 and 2, respectively. As the read current flows leftward through channel 2 along the x-axis, the AHE voltage in channel 2 is inverted. For the MDs of both channels pointing in the same ± z directions, the AHE voltage of channel 2 is also inverted, leading to V_s_ of ± 3 mV. The AHE voltages are monitored by the read current flowing through the channels, where the read voltage of ± 1 V provides the read current, as illustrated in Fig. 5. The logic outputs set to ‘T’ are determined by choosing a non-inverting comparator (V_s_ > 2 mV) or inverting comparator (V_s_ < 2 mV). (Experimental results regarding the logic gate operation by the read current direction are provided in Supplementary Fig. S4). To further achieve complex functions in future real microchips, one possible approach is to connect multiple gates in our scheme by adjusting the V_cc_ voltage of the comparators. For example, by increasing the V_cc_ voltage from 2 to 20 V, it can be ensured that V_out_ and logic inputs are equal, achieving the desired cascading effect. However, it should be noted that the ground should also be raised by + 10 V to maintain the proper voltage levels. This condition seems to be necessary for the successful operation of the cascaded logic gates.

## Conclusions

We successfully implemented eight reconfigurable logic gates using SOT currents in the two-channel Hall bar architecture. Utilizing the MDs of the two-channel, multiple logic gates are implemented using different SOT current flows induced by the same voltage-amplitude inputs. The non-volatile nature of MDs eliminates the need for time synchronization, which is typically required in conventional logic devices. With proper manipulation of the external magnetic field and read current directions, diverse reconfigurability of the logic gates is achieved, including AND, NAND, NOR, OR, Conver INH, Conver IMP, INH and IMP. These gates are described based on the inputs, read current directions, and non-inverting or inverting comparator selection in a fixed magnetic field. Thus, our experimental findings may pave the way for the realization of reconfigurable spin-logic building blocks that can be integrated with future SOT-MRAM or currently available CMOS technologies, thereby enabling practical applications in the future. To further implement the magnetic field assistance required for SOT switching in a future circuit, one additional electrode will be fabricated to generate the Oersted field above multiple logic gates in the near future. The current logic gates are designed to be reconfigurable by the direction of the read current under the same magnetic field. Therefore, future multiple logic gates have advantages in terms of control of the range of magnetic field and reconfigurable operation under the one Oersted magnetic field generated by an electrode.

## Methods

### Sample fabrication

The deposition process was performed using magnetron sputtering at room temperature, with a base pressure below 7 × 10^–9^ Torr and an Ar pressure of 3 mTorr. The composition of the layers in the stacks was as follows: [Si/SiO_2_] substrate/W (2)/Co_20_Fe_60_B_20_ (1.1)/MgO (1) Ta (2), where the numbers in parentheses indicate the thickness of each layer in nanometers. To enhance the perpendicular magnetic anisotropy properties, a post-annealing step was carried out. A post-annealing was conducted at 350 °C for 30 min under vacuum conditions of < 1 × 10^–6^ Torr with a 3 T perpendicular magnetic field. Following the deposition, the stacks were spin-coated with AZ5214E image reversal photoresist. Subsequently, photolithography and Ar ion milling techniques were employed to pattern the stacks into a two-channel Hall bar with a width of 5 μm. To remove the hardening photoresist after the ion milling procedures, an oxygen plasma ashing process was conducted for 2 min, utilizing 80 W of radiofrequency power. Acetone was used to lift off the photoresist. A electrode of W with a thickness of 200 nm was deposited to connect to the two-channel Hall bar structure.

It would be better to note that the offset transverse resistance in Hall bar devices may cause its potential impact on logic operations due to imperfections in the device geometry; that is, the offset transverse resistance can introduce errors in the logic operation since the anomalous Hall effect voltage is used as the logic output. Thus, in our initial work, several measures have been taken to possibly reduce the offset transverse resistance by including a precise alignment of the device components, rounding the edges of the Hall bar and electrodes, and ensuring the purity of the device surface by fabricating the device at an extremely low base pressure of < 7 × 10^–9^ Torr. These measures are particularly essential for minimizing geometric imperfections and reducing the offset transverse resistance. In addition, when downscaled devices are considered, specific design strategies should be considered to further mitigate the offset transverse resistance. For example, one effective approach is to position the electrodes closer to the central region of the device. It is expected that this approach can minimize the impact of the offset transverse resistance by reducing the distance over which the transverse resistance develops. This positioning also can help concentrate the current flow in the central region and minimizes the influence of geometric imperfections on the measurement of Hall voltage. The second approach is to make precise alignment of the device components, rounding the edges of the device, ensuring the purity of the device’s surface to mitigate offset transverse resistance, particularly in downscaled devices.

### MOKE microscopy and electrical measurement

A custom-built MOKE microscopy system with out-of-plane and in-plane electromagnets was used to monitor the MDs necessary for logic operations. The +z and −z MDs were clearly identified by the contrast difference in the MOKE microscopy images. Seven electrical probes were integrated into the MOKE system to attain the SOT current-induced MD switching behavior, and three and four probes were connected to the voltage source and Hall voltage detection terminals, respectively. Anomalous Hall effect voltages were monitored using a Keithley 236 and KEITHLEY 2000 multimeter. Additionally, to synchronize the MOKE images with the anomalous Hall effect voltage signals, programmed MOKE images were captured immediately after the injection of each voltage pulse. Reconfigurable logic operations are performed by switching the probes between the voltage source, ground, and floating point using a Keithley 708A switching system.

## Supplementary Information

Supplementary Information.

## Data Availability

The datasets used and/or analyzed during the current study are available from the corresponding author upon reasonable request.
