# Supplementary material for: Non-volatile reconfigurable spin logic functions in a two-channel Hall bar by spin–orbit torque-based magnetic domains and directional read current
Source: Sci Rep. 2023 Jul 18;13:11600. doi: 10.1038/s41598-023-38580-1 (PMC10354089; doi:10.1038/s41598-023-38580-1)
Supplement: Supplementary file 1 — Supplementary Information. [file 41598_2023_38580_MOESM1_ESM.docx]

SUPPLEMENTARY INFORMATION

Correspondence and requests for materials should be addressed to J.P.H ([jphong@hanyang.ac.kr](mailto:jphong@hanyang.ac.kr))

**Non-volatile reconfigurable spin logic functions in a two-channel Hall bar by spin-orbit torque-based magnetic domains and directional read current.**

*JeongHun Shin^1^, Jeongwoo Seo^2^, Saegyoung Song^2^, WooJong Kim^1^, Da Seul Hyeon^2^, and JinPyo Hong^1,2,^**

^1^Division of Nanoscale Semiconductor Engineering, Hanyang University, Seoul 133-791, South Korea

^2^Novel Functional Materials and Device Laboratory, Research Institute of Natural Science, Department of Physics, Hanyang University, Seoul 133-791, Korea

**1. MOKE image of MD switched by all inputs in an initial state.**


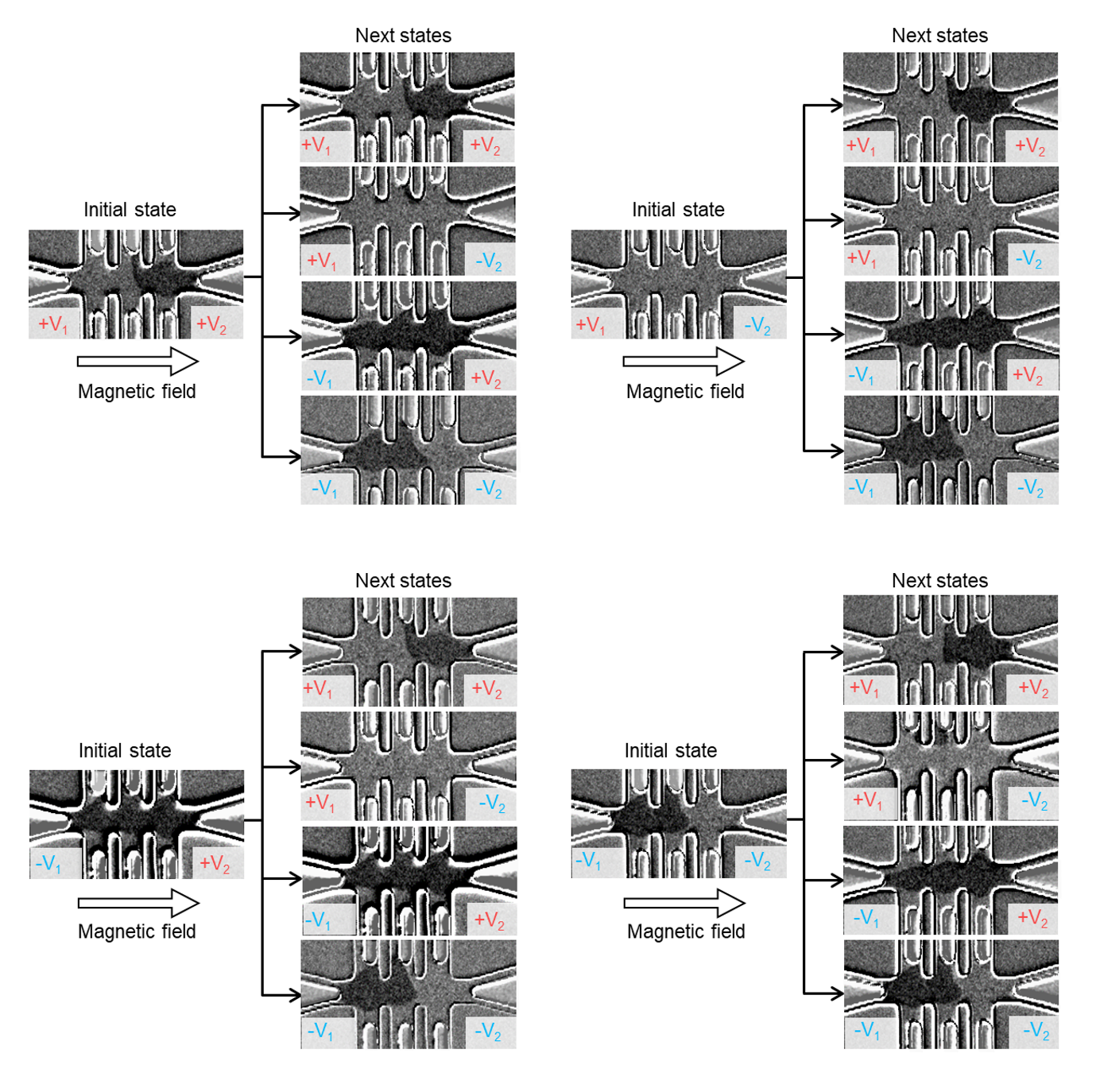


**Fig. S1.** MOKE image sequence from initial to the next consecutive states under V_1_ and V_2_ inputs. Various MOKE image sequences for the (+V_1_, +V_2_), (+V_1_, -V_2_), (-V_1_, +V_2_), and (-V_1_, -V_2_) initial states (left) and their subsequent states (right). The positive (red) and negative (blue) inputs of V_1_ and V_2_ reflect True and False, respectively. The arrows in each black line indicate all the possibilities for magnetic domain switching from the initial state to the next state by V_1_ and V_2_. Magnetic domain switching occurred in the presence of an external magnetic field of H_x_ = 100 Oe.

Fig. 3b in manuscript shows the MD switching results when the input voltages (+V_1_, +V_2_), (+V_1_, -V_2_), (-V_1_, +V_2_), and (-V_1_, -V_2_) are sequentially applied. Logic gate operations do not occur sequentially. Therefore, Fig. S2 illustrates the MD state-switching results from the initial states to all the different states. The initial state (up, down) on the left was set by applying inputs (+V_1_ and +V_2_). The other initial states are set up by applying inputs of (+V_1_, -V_2_), (-V_1_, +V_2_), and (-V_1_, -V_2_), respectively. The next states are switched by applying logic gate inputs (+V_1_, +V_2_), (+V_1_, -V_2_), (-V_1_, +V_2_), and (-V_1_, -V_2_). The MD switching results were confirmed using the MOKE image and were sufficiently stable to operate the logic gates. Inputs with voltage amplitudes of 10 V inducing a current density J_read_ = 1.0 x 10^7^ A/cm^2^ and a pulse duration of 100 ms were used at an external magnetic field of H_x_ = 100 Oe.

**2. Inverting comparator circuit and V_out_ result.**


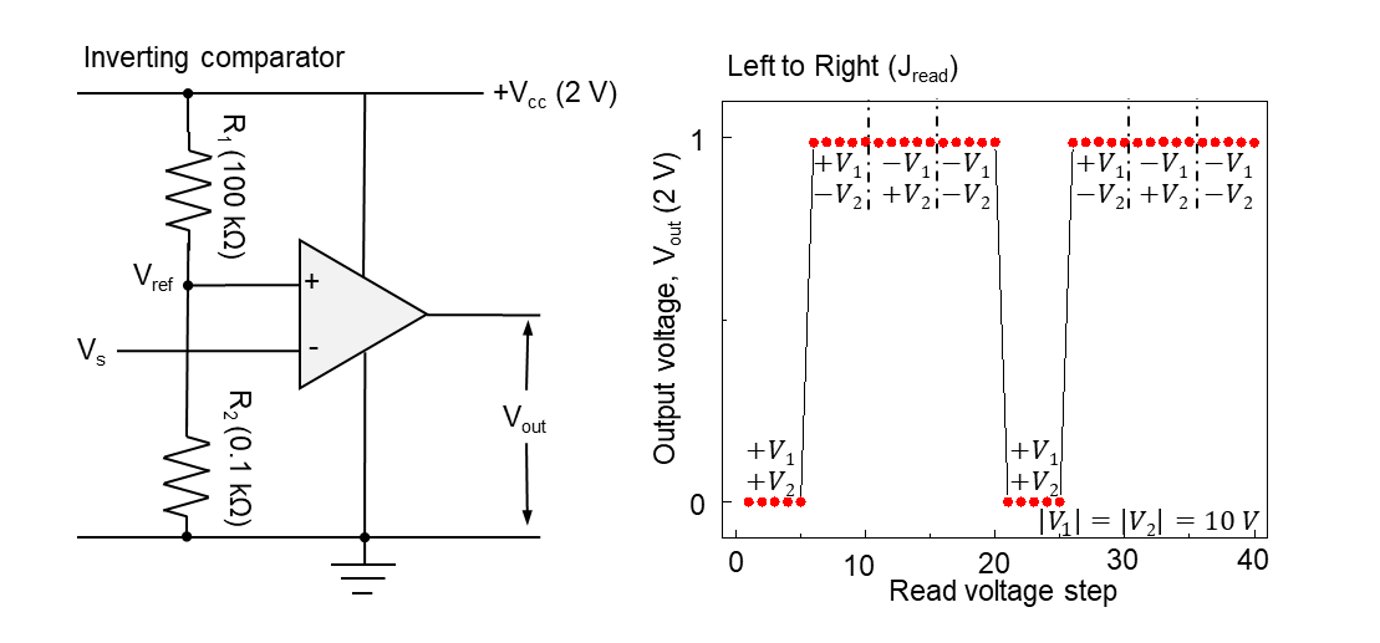


**Fig. S2.** Comparator circuit schematic and V_out_ curves by inverting comparator. Op-amp comparator circuits with inverting configurations, where the circuit uses a comparison voltage V_ref_ of 2 mV to produce various logic outputs V_out_, V_out_ in an inverting comparator circuit. The input voltages of both |𝑉_1_| and |𝑉_2_| in each scheme have the same amplitude of 10 V and are used to switch the MD states. The positive and negative amplitudes of 𝑉_1_ and 𝑉_2_ correspond to the True and False, respectively. The read current, measured from left to right is proportional to the voltage difference between the two inputs.

Fig. S2. shows an inverting comparator circuit and operation as a NAND gate. V_ref_ is connected to the positive terminal of the op-amp, while V_s_ is connected to the negative terminal in the inverting comparator. For example, when Vs is larger than V_ref_, V_out_ is reduced to 0 V. When V_s_ is smaller than V_ref_, V_out_ is amplified to 2 V. The two-channel Hall bar can implement a NAND gate based on the resulting V_out_ through the inverting comparator.

1. **Scaling of V_s_ with incremental read voltage amplitude**


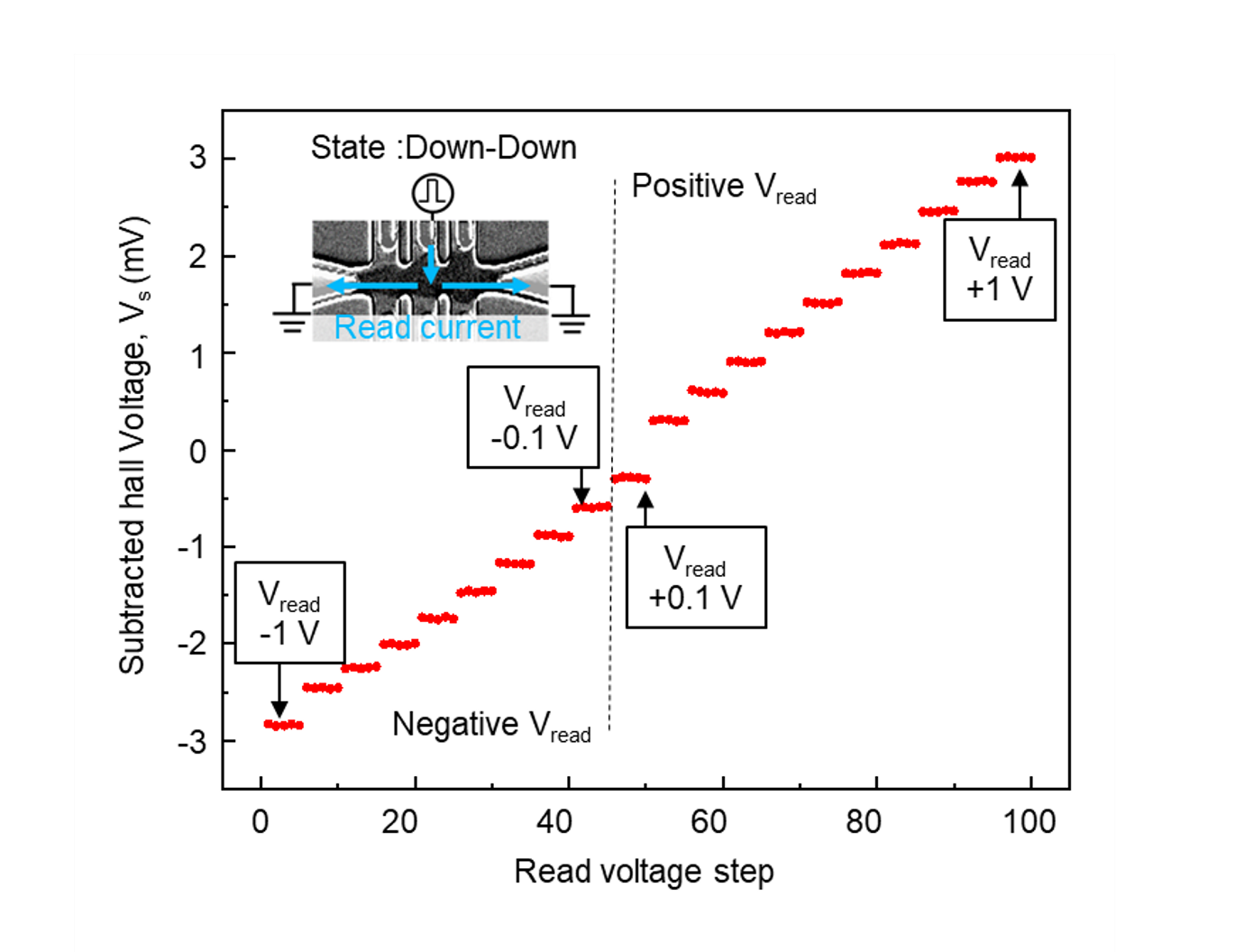


**Fig. S3.** Subtracted Hall voltage according to read voltage amplitude. The V_read_ amplitude was varied from -1 to 1 V in increments of 0.1 V for each set of five reading voltage steps. The corresponding V_s_ values were measured and plotted against V_read_ for each step, with the voltage increasing by 0.3 mV per V_read_ increment. The results show a linear relationship between V_s_ and V_read_ with a positive slope for positive V_read_ values and a negative slope for negative V_read_ values; that is, the polarity of V_s_ changed according to the polarity of V_read_.

Fig. S3 demonstrates the relationship between the subtracted Hall voltage (V_s_) and the read voltage amplitude (V_read_). The V_read_ amplitude is systematically varied from -1 V to 1 V in increments of 0.1 V, and for each set of five reading voltage steps, the corresponding V_s_ values are measured and plotted against V_read_. The V_s_ increments is set at 0.3 mV per V_read_ increment. Additionally, Fig. S3 shows the simultaneous opposite direction of the current flow. For example, when a V_s_ of +3 mV is applied, it indicates that the currents in the two channels flow to the left and right, respectively. When a read voltage of 1 V is applied, the following scenarios can be considered:

1) If the current flows through only one channel, the resulting V_s_ is the Hall voltage of that specific channel, which would be ±1.5 mV.

2) If the current flows through the two channels in the same direction, the resulting V_s_ is obtained by subtracting the identical Hall voltages of the two channels, resulting in ±0 mV.

3) If the current flows to the left and right, the V_s_ value measures +3 mV by subtracting the opposite values of the two channels. For instance, the anomalous Hall voltage of channel 1 is +1.5 mV when the current flows to the left, while channel 2 exhibits -1.5 mV when the current flows to the right. Thus, the V_s_ value of +3 mV indicates that the currents in the two channels flow to the left and right, respectively.

**4. Logic gate behaviors with read current directions.**


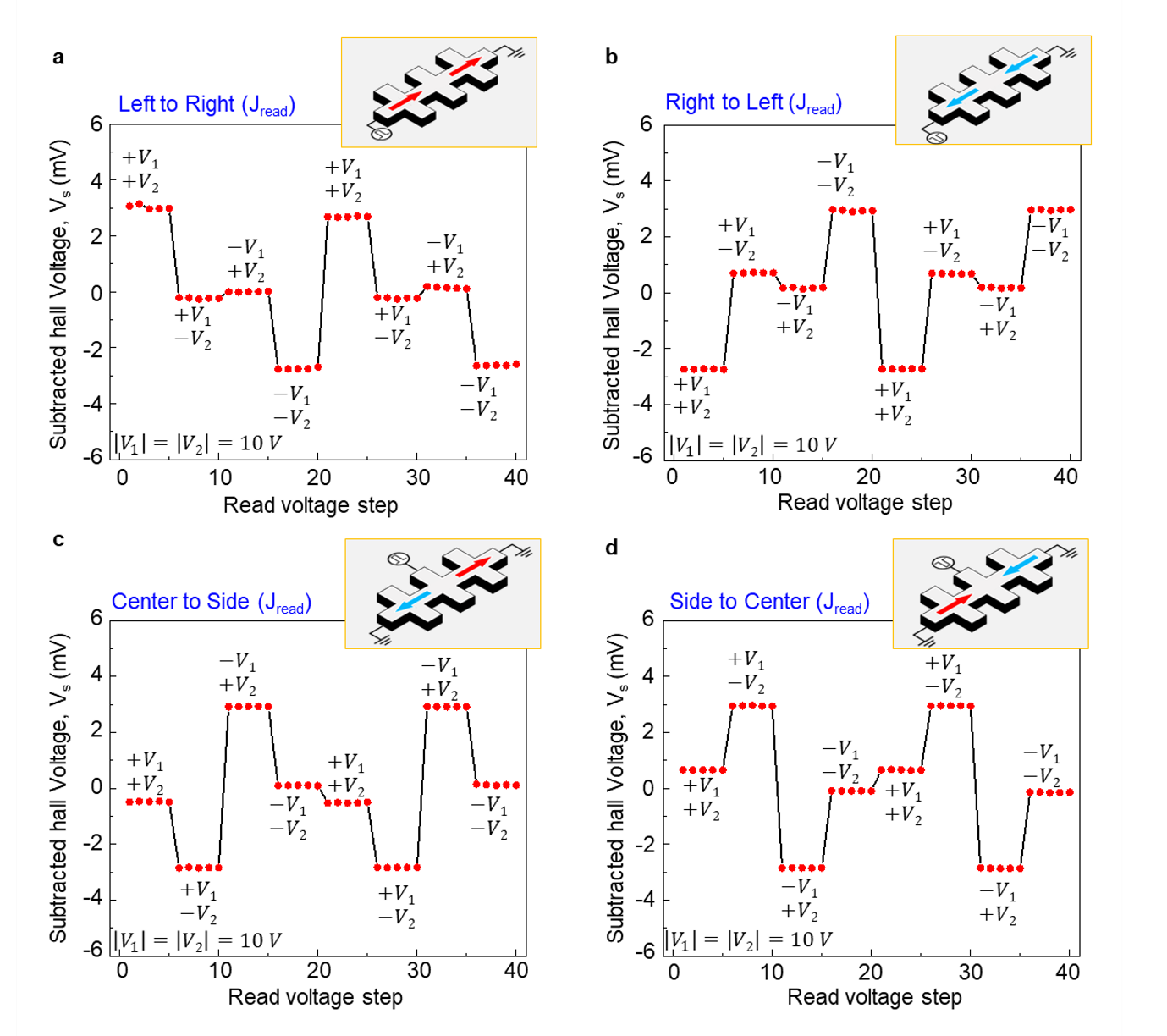


**Fig. S4.** Experimentally determined V_s_ curves under different read current directions. The variation in V_s_ was monitored under different read-current directions, with schematic illustrations of the read-current directions given above each graph. a) V_s_ under right-right read currents in both channels. b) V_s_ under left-read current in both channels. c) V_s_ under left current in Channel 1 and right current in Channel 2. d) V_s_ under the right current in Channel 1 and left current in Channel 2. In each case, the same 10 V amplitudes of V_1_ and V_2_ were used, indicating the voltages necessary to switch the MD states, and the positive and negative amplitudes reflect the True and False states, respectively.
